# The benefits of extrinsic ligament release for potentially hemodynamically unstable pancreaticoduodenal arcade aneurysm with median arcuate ligament syndrome: a case report

**DOI:** 10.1186/s12893-019-0514-8

**Published:** 2019-05-17

**Authors:** Takero Terayama, Yoshihiro Tanaka, Shigeyoshi Soga, Hironori Tsujimoto, Yuya Yoshimura, Yasumasa Sekine, Shinji Akitomi, Hisashi Ikeuchi

**Affiliations:** 1 Department of Traumatology and Critical Care Medicine, National Defense Medical College Hospital, Namiki 3-2, Tokorozawa, Saitama, 359-8513 Japan; 2 Department of Radiology, National Defense Medical College Hospital, Namiki 3-2, Tokorozawa, Saitama, 359-8513 Japan; 3 Department of Surgery, National Defense Medical College Hospital, Namiki 3-2, Tokorozawa, Saitama, 359-8513 Japan

**Keywords:** Pancreaticoduodenal artery aneurysm, Median arcuate ligament syndrome, Extrinsic median arcuate ligament release, Pancreaticoduodenal arcade, Case report

## Abstract

**Background:**

A pancreaticoduodenal artery aneurysm (PDAA) occurring in close association with median arcuate ligament syndrome (MALS) is rare. A surgical procedure, such as median arcuate ligament (MAL) release, should be considered in such cases, but the operative criteria remain unknown. In this study, we reported an extremely rare case of PDAA with periarteritis nodosa (PAN) and MALS.

**Case presentation:**

A 60-year-old man was transferred to our department with sudden onset of abdominal pain. We initially diagnosed his condition as a PDAA rupture with MALS based on enhanced computed tomography (CT). We promptly performed transcatheter arterial embolization (TAE) of PDAA, and the angiogram showed stagnant contrast agent in the celiac trunk, indicating total celiac artery occlusion. Follow-up enhanced CT three weeks after the first TAE clearly demonstrated newly formed, multiple aneurysms in the pancreaticoduodenal arcade and the hepatic artery. These findings indicated a systemic disorder, such as PAN or segmental arterial mediolysis, as the underlying cause. Therefore, we started corticosteroid therapy and performed diagnostic angiography to clarify the celiac artery’s patency. Contrary to the initial angiography, the second angiography showed sustained blood flow in the celiac artery. Nevertheless, we performed both extrinsic MAL release and consecutive TAE because of the risk of multiple aneurysms rupturing due to an uncontrolled systemic disorder and consequent hepatic ischemia. The patient had no episode of recurrence until one year of follow-up.

**Conclusions:**

It is important to evaluate risk for hemodynamically unstable events to decide the best treatment strategy for MALS.

## Background

Median arcuate ligament syndrome (MALS) is caused by a stenosis or occlusion of the celiac trunk by the median arcuate ligament (MAL). In response to the high arterial resistance caused by stenosis or celiac artery occlusion in MALS, pancreaticoduodenal artery aneurysm (PDAA) frequently occurs because of increased collateral blood flow through the pancreaticoduodenal arcade from the superior mesenteric artery (SMA).

Depending on the aneurysm’s location, extrinsic MAL release from the celiac trunk has to be considered before any intervention because liver ischemia can sometimes occur as a complication of embolizing aneurysms. However, PDAA caused by MALS is such a rare disease that no therapeutic guideline or criteria for MAL release has been established.

Here, we report a rare case of ruptured PDAA with MALS, in which laparoscopic MAL release was effective despite maintenance of substantial celiac blood flow on preoperative angiography. We emphasized the importance of establishing therapeutic criteria for PDAAs with MALS.

## Case presentation

A 60-year-old man was transferred to our department with sudden onset of abdominal pain. He had a past medical history of duodenal ulcer and paroxysmal atrial fibrillation, and had taken rivaroxaban. He had no allergy and no family medical history.

Physical examination showed initial findings of Glasgow coma scale, E4V5M6; blood pressure, 85/66 mmHg; respiratory rate, 25 /min; and peripheral oxygen saturation (SpO_2_), 100% at 6 L/min of oxygen by reservoir mask. He complained of sustained upper quadrant pain with abdominal guarding. Initial enhanced computed tomography (CT) demonstrated extravasation from the posterior inferior pancreaticoduodenal artery and celiac trunk stenosis. Thereafter, we diagnosed him with PDAA rupture due to MALS.

**Fig. 1:**
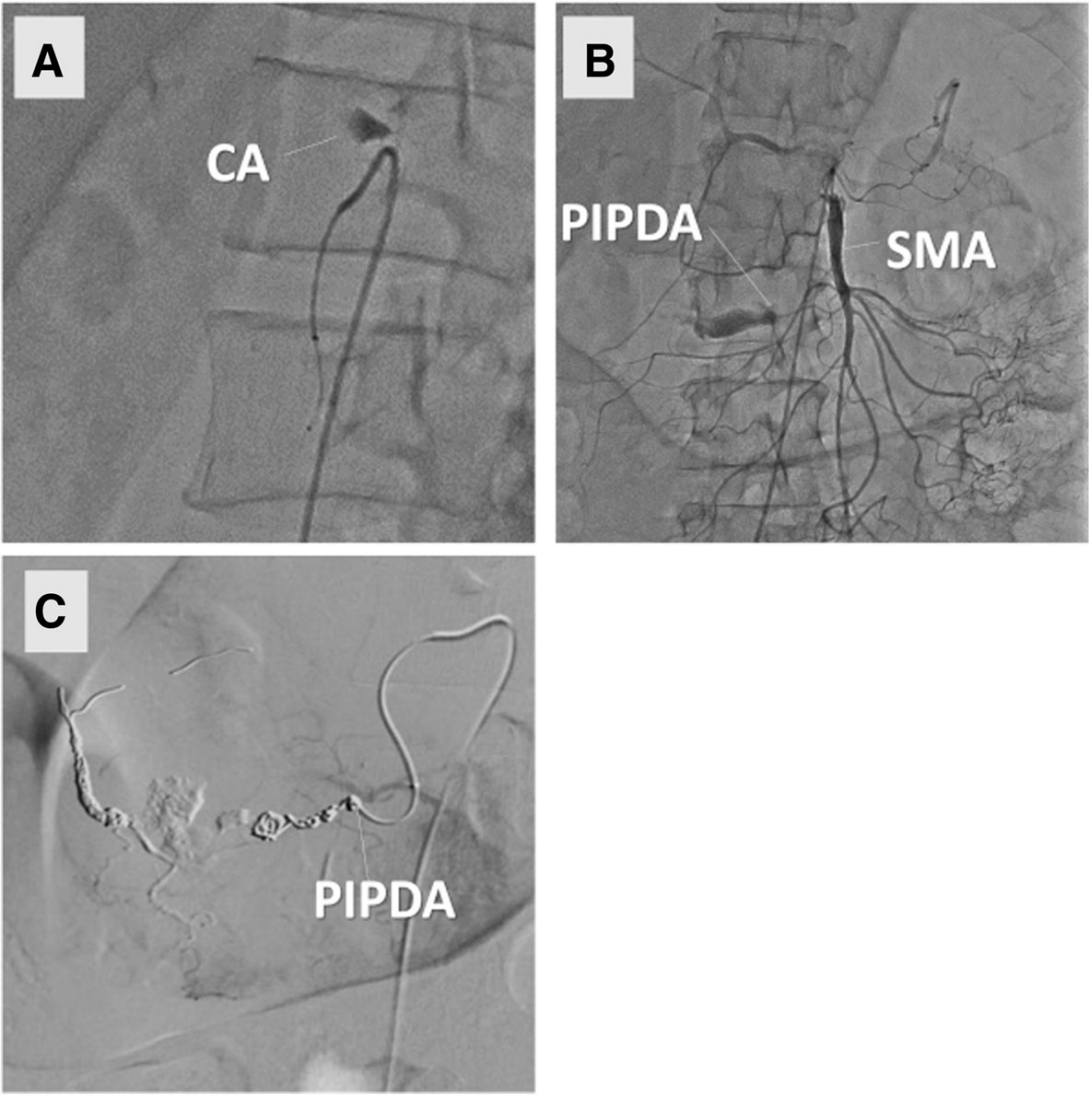
Transcatheter arterial embolization for a ruptured pancreaticoduodenal artery aneurysm with median arcuate ligament syndrome. **a** The stagnant contrast agent in the celiac trunk indicates total occlusion of the CA. **b** Digital subtraction angiography of the SMA shows retrograde blood flow from the PIPDA to the CA. **c** Embolization of the PIPDA aneurysm is performed using both coil and N-butyl-2-cyanoacrylate. CA, celiac artery; PIPDA, posterior inferior pancreaticoduodenal artery; SMA, superior mesenteric artery

First, an urgent transcatheter arterial embolization (TAE) was performed (Fig. 1). The PIPDA was selectively catheterized through the SMA, and embolization was performed using coils and N-butyl-2cyanoacrylate. The angiography at this time demonstrated both retrograde blood flow from the PIPDA to the celiac artery and stagnant contrast agent in the celiac trunk, indicating total celiac artery occlusion. The patient was admitted to the intensive care unit (ICU) because of some severe complications such as acute kidney injury, acidosis, and coagulopathy due to hemorrhagic shock. Continuous hemodiafiltration, intubation, and blood transfusion had been required in the ICU.

**Fig. 2:**
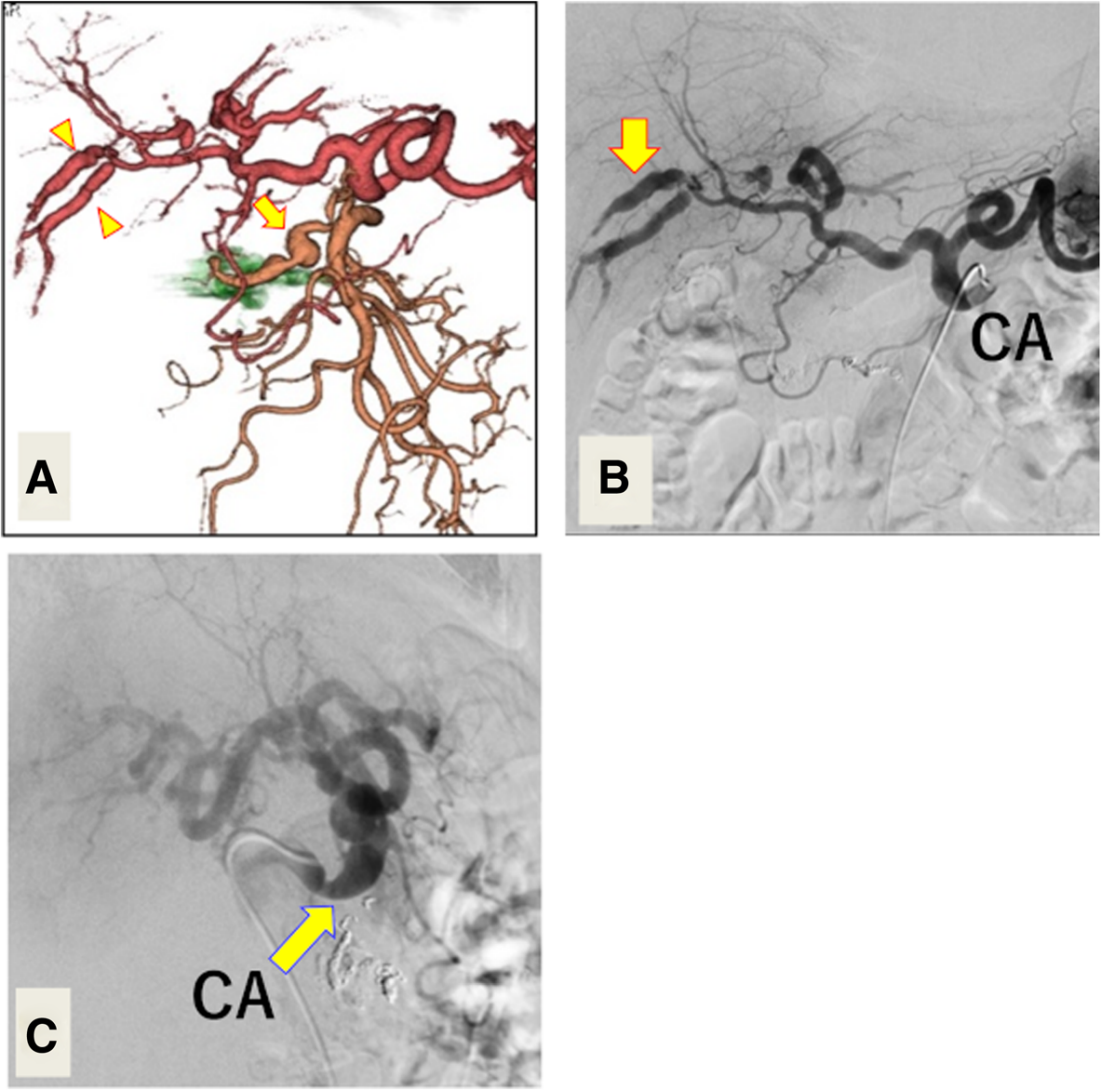
Enhanced computed tomography and diagnostic angiography performed prior to the second transcatheter arterial embolization. **a** Multiplanar reformatted image of the follow-up enhanced computed tomography on day 21 shows transverse pancreatic artery aneurysm (arrow) and hepatic artery aneurysms (arrowheads). **b** Diagnostic angiography on day 24 demonstrates sustained blood flow in the CA hepatic artery aneurysms (arrow). **c** Sagittal view of the angiography demonstrates severe stenosis of the CA (arrow). CA, celiac artery

The patient was followed-up with enhanced CT every week. Follow-up CT on day 21 after admission (Fig. 2a) demonstrated newly formed multiple aneurysms in the transverse pancreatic artery, hepatic artery, great pancreatic artery, and right renal artery; notably, the hepatic artery enlargement was bead-like.

Because of the high rupture risk for the aneurysms in the both the transverse pancreatic and hepatic arteries, a second angiography was performed to evaluate the possibility of liver ischemia after an additional TAE of the pancreaticoduodenal arcade. Unexpectedly, the angiogram on both expiratory and inspiratory phases revealed substantially preserved blood flow in the celiac trunk and hepatic artery aneurysms (Fig. 2b and c). Based on these findings, we postulated that hemorrhagic shock-induced vasospasm caused bloodstream changes during the initial angiography or that a systemic vascular disorder, such as periarteritis nodosa (PAN) or segmental arterial mediolysis (SAM), may be involved in pathophysiologic mechanisms of these events. Although the PAN diagnosis was uncertain, we initiated pulse steroid therapy with methylprednisolone at 1000 mg/day for three days.

We decided to perform extrinsic MAL release before additional pancreaticoduodenal arcade TAE, which was considered safe without causing liver ischemia based on the findings of both the first and second angiography. The pancreaticoduodenal arcade aneurysm was at high risk for rupture irrespective of the size. The hepatic artery aneurysm was also at high risk. Additional rupture would result in vasospasm of celiac artery and decrease of the flow and result in liver ischemia. Therefore, we performed a laparoscopic MAL release on day 29. Simultaneous excision biopsy of the greater omentum showed no diagnostic pathologic findings, except for slight vasculitis.

**Fig. 3:**
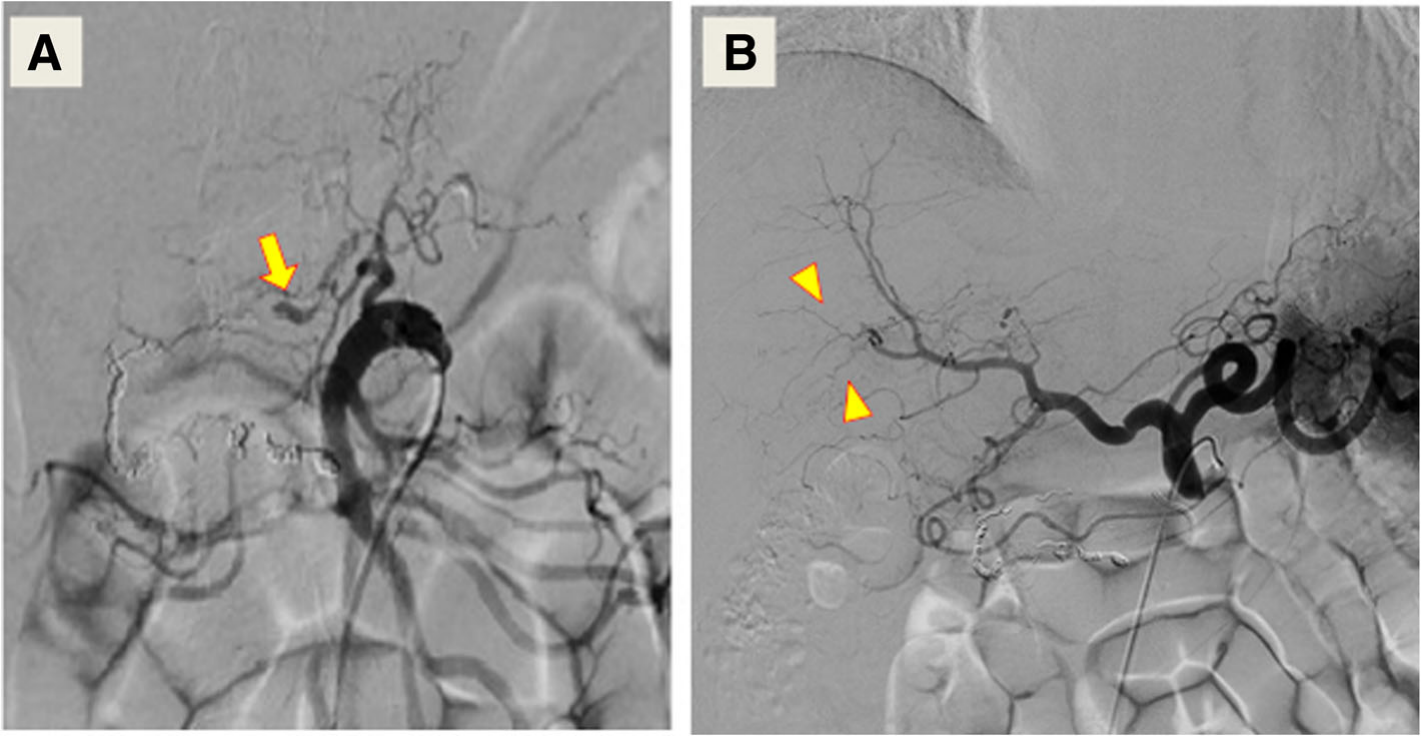
Angiography performed on day 35. **a** There is total occlusion of the transverse pancreatic artery aneurysm (arrow). **b** The hepatic artery aneurysms resolved drastically after median arcuate ligament release (arrowheads)

On day 35, the second TAE was planned. Contrary to our prediction, digital subtraction angiography demonstrated spontaneous occlusion of the transverse pancreatic artery and marked resolution of the other aneurysms, including the hepatic artery aneurysms (Fig. 3). Therefore, TAE was not performed. Although the blood examination did not fully satisfy the criteria for PAN, the patient received additional immunosuppressive therapy with oral prednisolone (60 mg/day) and intravenous cyclophosphamide (500 mg/day), which decreased the C-reactive protein (CRP) level to below 0.03 mg/dL. CRP level was maintained above 3.0 mg/dL until the therapy. For more than one year of follow-up, the patient remained recurrence-free and had no complaint about quality of life after discharge, even though hyperglycemia due to prednisolone required oral medication. The patient sent us a letter saying that he was satisfied with his current condition and had no complaint about our treatment.

## Discussion and conclusions

This was a rare case report on a ruptured PDAA with MALS and a probable systemic disease. MALS, which was first reported in 1963, results from anatomical compression of the celiac artery by the MAL. Celiac trunk stenosis or occlusion by the MAL increases the compensatory collateral blood flow from the SMA through the pancreaticoduodenal arcade. This hemodynamic change can induce both pressure and volume overload to the collateral vessel walls, finally resulting in aneurysm formation [1]. The relationship between MALS and PDAAs is well known, but the incidence of PDAA is uncommon and accounts for only 2% of all visceral artery aneurysms [2].

The probability of rupture is much higher for a pancreaticoduodenal arcade aneurysm than for any other aneurysms because a pancreaticoduodenal arcade aneurysm tends to rupture regardless of size. Pancreaticoduodenal arcade consists of pancreaticoduodenal artery, transverse pancreatic artery, and so on. Therefore, early therapy is recommended for all aneurysms as soon as they are detected. Recently, endovascular repair was established as an initial therapy for aneurysms. Depending on the aneurysms’ locations, liver ischemia from a sudden decrease in blood supply is one of the major complications of TAE. In cases of PDAA with MALS, the main blood supply to the liver is from a collateral blood flow from the arcade. Therefore, if the blood flow through the celiac artery is insufficient, it is rational to perform extrinsic release of the celiac trunk before TAE of the arcade [3]. In our case, however, we performed a laparoscopic MAL release despite adequate blood flow through the celiac artery on angiography.

We decided on the above-mentioned approach for two reasons. First, the change in the celiac trunk blood flow, demonstrated in the initial TAE and in the second angiography, must have been caused by vasospasm due to hemorrhagic shock. Second, the risk for aneurysmal rupture would remain high if the suspected systemic disease (i.e., PAN) was not controlled. Therefore, we deduced that unless MAL release was performed before pancreaticoduodenal arcade TAE, an aneurysmal rupture may lead to the same vasospasm and, possibly, fatal liver ischemia. Extrinsic MAL release was a beneficial and safe strategy in this case.

There is no evidence on the necessary period for sufficient collateral blood flow to develop. PDAA is so rare that definitive criteria for MAL release are not yet available. To delineate these criteria, it may be necessary to consider the relationship between collateral blood flow occlusion and liver ischemia, including the growth speed of sufficient collaterals. This case strongly suggested that extrinsic MAL release must be considered in patients at risk for hemodynamic instability. Conversely, some have reported that extrinsic MAL release may be unnecessary. Sgroi et al. reported three cases without celiac artery revascularization, stating that revascularization may be unnecessary in asymptomatic patients [4]. Shibata et al. demonstrated no significant change in the blood pressure of the common hepatic artery between the pre-TAE and post-TAE phases [5]. However, these two reports did not refer to the risk for hemodynamic instability previously mentioned. Further investigations will be necessary to resolve controversies such as liver ischemia.

There is also insufficient evidence with regard to long-term outcome of celiac trunk revascularization with both extrinsic ligament release and stenting; therefore, we chose the former strategy considering our experiences in this case.

In conclusion, it is important to evaluate risk for hemodynamically unstable events to decide the best treatment strategy for MALS.

## Abbreviations

CRP: C-reactive protein
CT: Computed tomography
ICU: Intensive care unit
MAL: Median arcuate ligament
MALS: Median arcuate ligament syndrome
PAN: Periarteritis nodosa
PDAA: Pancreaticoduodenal artery aneurysm
PIPDA: Posterior inferior pancreaticoduodenal artery
SAM: Segmental arterial mediolysis
SMA: Superior mesenteric artery
TAE: Transcatheter arterial embolization
